# Benefits of Interventional Telemonitoring on Survival and Unplanned Hospitalization in Patients With Chronic Heart Failure

**DOI:** 10.3389/fcvm.2022.943778

**Published:** 2022-07-14

**Authors:** Michel Galinier, Romain Itier, Anthony Matta, Montse Massot, Pauline Fournier, Ghislaine Galtier, Sandrine Ayot, Vanessa Nader, Max Rene, Laurent Lecourt, Jerome Roncalli

**Affiliations:** ^1^Heart Failure Unit, Department of Cardiology, Institute CARDIOMET, University Hospital of Toulouse, Toulouse, France; ^2^CDM e-Health, Jouy-en-Josas, France; ^3^Air Liquide Santé International, Gentilly, France

**Keywords:** heart failure, telemonitoring, hospitalization, OSICAT, ETAPES

## Abstract

**Aims:**

To assess the effect of interventional specialized telemonitoring (ITM) compared to standard telemonitoring (STM) and standard of care (SC) on preventing all causes of death, cardiovascular mortality and unplanned hospitalization in heart failure (HF) patients.

**Methods:**

We compared outcomes in three groups of HF patients followed by different modalities: SC, STM and ITM. The telemonitoring was performed by the specialized HF-cardiology staff at Toulouse University Hospital. All patients were followed with the same manner including daily weight monitoring using on-line scales, self-monitoring and reporting symptoms *via* a device. The difference between groups was in the management of the received alerts. In STM-group, patients were contacted by a member of telemedical center and the main responsibility for patient's therapy was taken by their primary care physicians while in the ITM-group, a cardiologist intervenes immediately in case of alerts for diuretic dose adjustment or escalation therapy or programmed hospitalization if necessary. Outcomes were compared between the three study groups and Kaplan-Meier analysis was performed.

**Results:**

Four hundred fourteen HF-patients derived from two French cohorts (OSICAT and ETAPES) were included in this study and subsequently enrolled in the following three groups: ITM-group (*n* = 220), STM-group (*n* = 99), and SC-group (*n* = 95). During the mean follow-up period of 341 days, there were significantly fewer primary endpoints like unplanned hospitalization (13.6 vs. 34.3 vs. 36.8%, *p* < 0.05), all-causes of death (4.5 vs. 20.2 vs. 16.8%, *p* < 0.05) and cardiovascular mortality (3.2 vs. 15.2 vs. 8.4%, *p* < 0.05) in the ITM-group. The multivariable logistic regression revealed a significant negative association between the ITM and unplanned hospitalization [OR = 0.303 95% CI (0.165–0.555), *p* < 0.001) and all-causes of death [OR = 0.255 95% CI (0.103–0.628), *p* = 0.003], respectively. Kaplan Meier and log rank test showed significant difference in median event-free survival in favor of ITM-group.

**Conclusions:**

In the ITM follow-up HF group, delivered by a cardiology team, the rate of unplanned hospitalization and all-causes of death are lower than SC or STM.

## Introduction

Improving care delivery in the community is a critical challenge for improving the management of patients with chronic heart failure (HF). Telemonitoring associated with therapeutic support, may be a relevant answer to this challenge, by empowering patients to be responsible for their own monitoring. Meta-analyses based on early trials have demonstrated a decrease in the risk of hospitalization for heart failure (HF) and in mortality in patients managed by telemonitoring (1, 2) while findings from recent studies have shown conflicting results. Indeed, the TIM-HF2 study (3) has revealed a decrease in mortality rate unlike the TELE-HF (4), TIM-HF (5), BEAT-HF (6) and OSICAT (7) studies, respectively.

There may be several explanations for these controversial findings. The value of study participant characteristics seems to be important. The most benefit from telemonitoring has been observed in patients with severe HF (NYHA stage III or IV) and compliant patients, whereas depression seems to compromise benefits (3, 7). The advancement of monitored parameters is less likely significant. Most frequently, monitoring consists of weight measurement and symptoms assessment. Monitoring of electrocardiographic data may be relevant, but hemodynamic monitoring [measurement of thoracic impedance (8, 9) or pulmonary pressures (10) with equipment at the patient's home] has given disappointing results. How to respond to alert could be a determining element; for example, in the TIM-HF 2 study (3) a quasi-permanent monitoring delivered by the cardiology team has proved to be very effective, whereas this was not the case in the OSICAT trial (7), where the patient had to consult his general practitioner in case of an alert.

In order to explore the impact of specialized telemonitoring, we aim to assess the effect of interventional specialized Telemonitoring (ITM) compared to standard telemonitoring (STM) and standard of care (SC) on preventing all causes of death, cardiovascular mortality and unplanned hospitalization in HF patients.

## Methods

### Study Design and Population

The present study was conducted on HF-patients from the French Optimization of the ambulatory monitoring for patients with heart failure by tele-cardiology (OSICAT study) and the French Telemedicine experiments for the improvement of health care paths (ETAPES program). The OSICAT study, conducted between 2013 and 2016, involved patients hospitalized for acute HF in the preceding 12 months who were randomized to non-medical telemonitoring (TM) or standard of care (SC) and followed-up for 18 months. The French ETAPES program developed by the French public health authorities is investigating the use of interventional telemedicine in improving healthcare pathways in several patient groups, including those with chronic HF. This program initiated in 2018 for a 3-year period has been extended to 2022 to more clearly evaluate and define the role of telemedicine. Patients from the ETAPES program have been included between March 2018 and October 2020 and followed for at least 6 months (11–13).

Thus, patients with symptomatic chronic heart failure (NYHA stage ≥II) who had been hospitalized in the previous 12 months for acute decompensation of HF and who presented a brain natriuretic peptide (BNP) level ≥100 pg/mL or a NT-pro-BNP level of ≥300 pg/ml (OSICAT trial) or ≥1,000 pg/mL (ETAPES program) have been included in this study. In March 2020, the eligibility criteria for the ETAPES program were broadened due to the COVID19 pandemic to concern all patients with chronic heart failure. Patients with advanced neurocognitive disorders were excluded from this study because they are not able to report the symptoms and adhere to a regular monitoring.

We compared outcomes between the three groups of patients followed by different modalities: the standard of care HF-patients conventionally followed by their general physicians (SC-group), HF-patients followed with standard telemonitoring (STM-group), and HF-patients followed by interventional telemonitoring (ITM-group). The first two groups were selected from the OSICAT trial and the main responsibility for patient's therapy was taken by their primary care physicians. In STM-group, patients were regularly contacted by a nurse of telemedical center on a monthly basis and in case of alerts to request a medical visit of their general physicians. However, the ITM-group was selected from the ETAPES program, and patients were regularly contacted by a trained HF-nurse for therapeutic education. In case of alerts, a cardiologist intervenes immediately for diuretic dose adjustment, escalation therapy and programmed hospitalization if necessary. The ITM-group was subsequently segregated into two sub-groups based on sacubitril/valsartan (ARNI) intake view the progression of HF medical treatment overtime (14). This variation ensued from the difference in time of the inclusion period between the two cohorts. This step was performed to attenuate the potential bias related to ARNI intake in the ITM-group which was not observed in the SC- and STM-groups.

The primary endpoint was to assess all-causes of mortality, cardiovascular mortality and unplanned hospitalizations in each group.

### Telemonitoring Procedures

All patients were followed with the same manner including daily weight monitoring using on-line scales, self-monitoring and reporting symptoms *via* a device. The difference between groups was in the management of the received alerts. Input from patients was remotely manipulated during working hours over 5 days per week. An alert was generated in case of weight gain of ≥3 kg over 2 days or ≥2 kg over 5 days or in case of worsening of at least three symptoms (nocturnal dyspnoea, orthopnoea, cough, oedema or fatigue) on the same day or worsening of at least two symptoms on three consecutive days. In the STM-group derived from OSICAT trial, nurses called the patients in case of an alert to request general practitioner consultation in contrast to ITM-group derived from the ETAPES program where the nurse contacted the cardiology team to organize either urgent teleconsultation or face-to-face consultation usually ending with medical treatment adjustment.

Self-monitoring was associated with therapeutic education provided over the phone which focus on explanation about the symptoms and signs of decompensation, importance of low-salt diet, physical activity and compliance with the medical treatment. This support was provided by specialized nurses trained in HF and therapeutic education.

### Statistical Analysis

Categorical variables were summarized as numbers and percentages while continuous variables as mean values and standard deviations. Continuous variables were compared using analysis of variance (ANOVA), and categorical variables using the chi^2^ or Fisher's exact tests, as appropriate. A stepwise logistic regression was conducted to assess the association between ITM on the one hand, and unplanned hospitalization, cardiovascular mortality and all cause of mortality, on the other. The regression was adjusted for all variables differing between the groups with a *p*-value <0.2 in the bivariate analyses. Outcomes were compared between the three study groups and presented as odds ratios (OR) with their 95% confidence intervals (95% CI), taking the standard of care group as the reference group. Hosmer-Lemeshow and omnibus tests were verified to test model fit.

Kaplan-Meier analysis was performed to estimate the cumulative unplanned hospitalization rate and all cause of mortality rate. The log rank test was used to compare the differences in hospitalization and mortality between the three groups. A subgroup analysis of the ITM-group was performed in patients who were or were not treated with sacubitril-valsartan, in order to evaluate the impact of ITM independently of treatment. A two-sided *p*-value of <0.05 was considered statistically significant. All statistical analyses were carried out by using SPSS statistics 20.

## Results

### Study Participants

Overall, 414 HF-patients were enrolled in this study accounting for 220 patients in the ITM-group, 99 patients in the STM-group and 95 patients in the SC-group. The mean age was of 63.45 years and 79.2% of patients were males with no significant difference in sex prevalence among study groups. Out of study participants, 8.9% have been only recruited by ambulatory process. Table 1 showed the baseline and demographic characteristics of the studied population.

**Table 1:** Baseline characteristics of studied population.

	**Studied population *N* = 414**	**SC-group *N* = 95**	**STM-group *N* = 99**	**ITM-group *N* = 220**	***P*-value**
Age	63.45 ± 13.98	66.24 ± 13.20	66.57 ± 12.78	60.84 ± 14.36	<0.05
Male sex	328 (79.2%)	75 (78.9%)	79 (79.8%)	174 (79.1%)	0.987
BMI (kg/m^2^)	26.92 ± 5.37	27.12 ± 5.54	26.90 ± 5.02	26.84 ± 5.47	0.917
Hypertension	170 (41.6%)	46 (48.4%)	48 (48.5%)	76 (35.3%)	0.027
Diabetes mellitus	108 (26.4%)	35 (36.8%)	31 (31.3%)	42 (19.5%)	0.03
Heart transplant	30 (7.2%)	6 (6.3%)	10 (10.1)	14 (6.4%)	0.454
Ambulatory	37 (8.9%)	1 (1.1%)	3 (3.0%)	33 (15.0%)	<0.05
Ischemic HF	171 (41.4%)	47 (50.0%)	46 (46.5%)	78 (35.4%)	0.028
LVEF (%)	30.21 ± 11.97	31.43 ± 12.79	33.33 ± 13.62	28.28 ± 10.40	0.005
NTproBNP (pg/ml)	6010 ± 8398	6205 ± 7588	7141 ± 11279	5522 ± 7367	0.932
Beta-blocker	340 (83.1%)	77 (81.1%)	76 (76.8%)	187 (87%)	0.067
(ACEi, ARB, ARNIs)	334 (81.7%)	74 (77.9%)	71 (71.7%)	189 (87.9%)	0.001
Furosemide	399 (96.4%)	90 (94.7%)	94 (94.9%)	215 (97.7%)	0.248
Ivabradine	48 (11.7%)	9 (9.5%)	4 (4.0%)	35 (16.3%)	0.005

*SC-group, standard of care group; STM-group, standard telemonitoring-group; ITM-group, interventional telemonitoring group; BMI, body mass index; HF, heart failure; LVEF, left ventricular ejection fraction; ACEi, Angiotensin converting enzyme inhibitor; ARB, Angiotensinogen 2 receptor antogaonist; ARNIs, Angiotensin receptor-neprilysin inhibitors*.

Cardiovascular risk factors like diabetes mellitus (19.5% in ITM-group vs. 31.3% in STM-group vs. 36.8% in SC-group) and arterial hypertension (35.3 vs. 48.5 vs. 48.4%) were significantly less common in the ITM-group. HF-patients in the ITM-group were also younger (60.84 vs. 66.57 vs. 66.24 years), but they expressed the lowest mean of left ventricular ejection fraction (28.28 vs. 33.33 vs. 31.43%). Compared to other groups, the proportion of HF-patients enrolled by ambulatory basis in the ITM-group was high (15 vs. 3 vs. 1.1%). No significant statistical difference regarding natriuretic peptide level has been detected between study groups.

As predicted, the proportion of patients treated with angiotensin receptor-neprilysin inhibitors (ARNIs), angiotensin II receptor blocker (ARB) or angiotensin converting enzyme inhibitor (ACEi) was remarkably higher in the ITM-group than that in STM- and SC-groups (87.9 vs. 71.7 vs. 77.9%, *p* < 0.05). No ARNIs intake was noted in STM-group and SC-group.

### Outcomes

Morbidity and mortality data observed after a mean follow-up of 344.91 days are presented in Table 2. In bivariate analyses, the prevalence of unplanned hospitalization was significantly lower in the ITM-group (13.6%) than that in the STM-group (34.3%) and SC-group (36.8%). In parallel, all-causes mortality rate was significantly lower in the ITM-group (4.5%) than that in the STM-group (20.2%) and SC-group (16.8%). Although, cardiovascular mortality rate was similarly lower in the ITM-group (3.2%) than that in the STM-group (15.2%) and SC-group (8.4%). The mean duration of follow-up in ITM-group, STM-group and SC-group were 276, 362, and 437 days, respectively.

**Table 2:** Observed outcomes in each group during the period of follow-up.

	**Studied population *N* = 414**	**SC-group *N* = 95**	**STM-group *N* = 99**	**ITM-group*N* = 220**	***P*-value**
Duration of follow-up (days)	341 ± 194	437 ± 190	362 ± 207	276 ± 163	<0.05
Unplanned hospitalization	99 (23.9%)	35 (36.8%)	34 (34.3%)	30 (13.6%)	<0.05
All-cause of mortality	46 (11.1%)	16 (16.8%)	20 (20.2%)	10 (4.5%)	<0.05
Cardiovascular mortality	30 (7.2%)	8 (8.4%)	15 (15.2%)	7 (3.2%)	<0.05

### Multivariate Analyses

**Figure 1:**
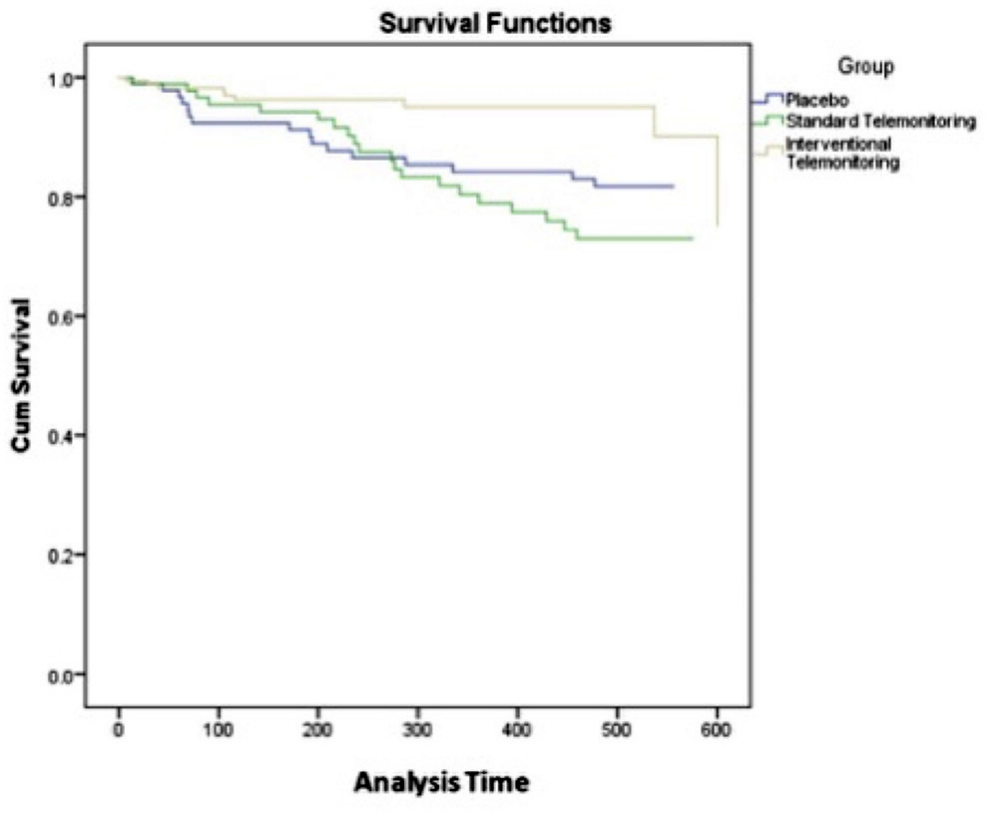
Kaplan-Meier analysis of survival at last follow-up (in days) for ITM-group vs. STM- and SC-groups. There was a significant difference in all-cause of mortality (*p* = 0.009).

The multivariate analyses adjusted for age, inclusion modality (outpatient or inpatient), etiology (ischaemic or non-ischaemic), cardiovascular risk factors (arterial hypertension and diabetes mellitus), mean ejection fraction and medical treatment have demonstrated a significant negative association between ITM and unplanned hospitalization [OR = 0.303 95% CI (0.165–0.555), *p* < 0.001] and all-causes of mortality [OR = 0.255 95% CI (0.103–0.628), *p* = 0.003] (Tables 3, 4). Multivariate analysis also trends toward a significant negative association between ITM and cardiovascular mortality [OR = 0.322 95% CI (0.103–1.012), *p* = 0.052] (Table 5). Lastly, the Kaplan Meier analyses revealed significantly different event free-survival regarding unplanned hospitalization (*p* = 0.049) and all-cause of mortality (log-rank test, *p* = 0.009) (Figure 1) in favor of ITM-group.

**Table 3:** Adjusted multivariate analysis investigating the association between interventional telemonitoring (ITM) and unplanned hospitalization compared to other study groups.

	**ORa**	**95% CI**	***P*-value**
Age	0.991	(0.970–1.012)	0.383
Hypertension	1.153	(0.691–1.922)	0.586
Diabetes mellitus	1.286	(0.741–2.234)	0.371
Ischemic-HF	1.144	(0.693–1.889)	0.599
LVEF	0.989	(0.967–1.011)	0.336
Ivabradine use	0.456	(0.173–1.200)	0.112
(ACEi, ARB, ARNIs)	0.544	(0.298–0.994)	0.048
Ambulatory process	0.969	(0.360–2.610)	0.950
**Groups**			
-STM-group	0.862	(0.472–1.574)	0.629
-ITM-group	0.303	(0.165–0.555)	<0.001

*STM-group, standard telemonitoring-group; ITM-group, interventional telemonitoring group; BMI, body mass index; HF, heart failure; LVEF, left ventricular ejection fraction; ACEi, Angiotensin converting enzyme inhibitor; ARB: Angiotensinogen 2 receptor antagonist; ARNIs: Angiotensin receptor-neprilysin inhibitors; ORa, adjusted odds ratios*.

**Table 4:** Adjusted multivariate analysis investigating the association between interventional telemonitoring (ITM) and all-cause of mortality compared to other study groups.

	**ORa**	**95% CI**	***P*-value**
Age	1.034	(1.002–1.067)	0.036
Hypertension	0.549	(0.270–1.117)	0.098
Diabetes mellitus	1.292	(0.613–2.724)	0.501
Ischemic-HF	0.894	(0.453–1.763)	0.746
LVEF	0.957	(0.926–0.988)	0.007
Ivabradine use	0.443	(0.094–2.082)	0.303
(ACEi, ARB, ARNIs)	0.452	(0.212–0.962)	0.039
Ambulatory process	0.968	(0.248–3.773)	0.963
**Groups**			
-STM-group	1.250	(0.584–2.675)	0.565
-ITM-group	0.255	(0.103–0.628)	0.003

*STM-group, standard telemonitoring-group; ITM-group, interventional telemonitoring group; BMI, body mass index; HF, heart failure; LVEF, left ventricular ejection fraction; ACEi, Angiotensin converting enzyme inhibitor; ARB: Angiotensinogen 2 receptor antagonist; ARNIs: Angiotensin receptor-neprilysin inhibitors; ORa, adjusted odds ratios*.

**Table 5:** Adjusted multivariate analysis investigating the association between interventional telemonitoring (ITM) and cardiovascular mortality compared to other study groups.

	**ORa**	**95% CI**	**P-value**
Age	1.036	(0.997–1.077)	0.069
Hypertension	0.615	(0.261–1.451)	0.267
Diabetes mellitus	1.047	(0.411–2.668)	0.923
Ischemic-HF	0.990	(0.437–2.241)	0.981
LVEF	0.927	(0.887–0.970)	0.001
Ivabradine use	0.309	(0.038–2.532)	0.274
(ACEi, ARB, ARNIs)	0.584	(0.226–1.508)	0.267
Ambulatory process	1.677	(0.405–6.933)	0.476
**Groups**			
-STM-group	1.986	(0.768–5.134)	0.157
-ITM-group	0.322	(0.103–1.012)	0.052

*STM-group, standard telemonitoring-group; ITM-group, interventional telemonitoring group; BMI, body mass index; HF, heart failure; LVEF, left ventricular ejection fraction; ACEi, Angiotensin converting enzyme inhibitor; ARB, Angiotensinogen 2 receptor antagonist; ARNIs, Angiotensin receptor-neprilysin inhibitors; ORa, adjusted odds ratios*.

### Subgroup Analysis

Despite that multivariate stepwise logistic regression analyses were adjusted on medical treatment differences (ivabradine, ARNIs, ACEi, ARB), we have segregated the ITM-group into two subgroups (ARNIs-ITM-group, *n* = 77 and NoARNIs-ITM-group, *n* = 143) according to ARNI intake which was not observed in STM- and SC-groups in order to investigate furthermore the impact of ITM after attenuating this potential bias. Then, the repeated multivariate analyses (SC-group, STM-group, ARNI-ITM group and NoARNI-ITM-group) have also demonstrated the negative association between ITM and unplanned hospitalization [OR = 0.293 95% CI (0.150–0.575), *p* < 0.001] and all-cause of mortality [OR = 0.264 95% CI (0.097–0.718), *p* = 0.009] Supplementary Tables 1, 2.

## Discussion

The main finding of this study was the positive effect of using ITM as follow-up modality on all-causes mortality rate and the prevalence of unplanned hospitalization in HF-patients compared to the conventional standard of care and telemonitoring. The low prevalence of cardiovascular events may limit the power of the study to detect a statistical difference regarding the cardiovascular mortality.

This study represents a *post hoc* analysis comparing HF-patients from OSICAT trial to those of ETAPES program. Both trials were conducted in the same department of Cardiology at the University Hospital of Toulouse, thereby promoting the homogeneity of telemonitoring technique (telemonitoring system implemented by CDM e-Health, an Air liquid affiliate), which consists of daily monitoring of weight and symptoms. In both groups, telemonitoring was associated with therapeutic support carried out by specialized nurses trained in therapeutic education and heart failure. The two types of telemonitoring share the designed algorithm for alerts management putting nurses on the frontline. Then, false alarms and low-level alerts are systematically filtered at this level. However, how to deal with the high-level alerts makes the ultimate difference. For STM, HF-patients have been contacted by the nurse to recommend consultation with their general healthcare givers while for ITM, nurses intervene to assess symptoms by phone call and scheduled urgent consultation with our cardiology staff. Diuretic dose adjustment, medical treatment upgrading and immediate hospitalization for critically ill patients constitute the main ensuing results from these cardiology staff interventions.

The speed, quality and efficiency of response to high level alerts seems the key explanation for the observed benefits of ITM independently from medical treatment. An early appropriate intervention either for diuretic dose adjustment in acutely decompensated HF-patients eventually reduces the need for hospitalization for intravenous diuretics or for immediate hospitalization in critically decompensated HF-patients significantly decreases the mortality rate. Data from literature have demonstrated the importance of early intervention and home-based intervention in congestive HF-patients (14). Noteworthy that the effectiveness of conventional telemonitoring depends on patient's willingness to consult their medical health practitioners and physician's compliance for making an urgent appointment. Thus, the high adherence to daily body weight measurement and symptoms assessment is the cornerstone element of success of telehealth programs (7). This study result was in line with the conclusion of a recently published study that showed a positive influence of telemedical interventional monitoring on depression in HF-patients and a better quality of life in the ITM-group compared to those receiving usual care (15). A significant difference in mean change from baseline level of the physical and mental component summary has been observed. Like our study, the ITM consists of permanent monitoring of blood pressure and body weight *via* a non-invasive device. However, the management of received alerts and the primary objective differ between these two studies. This later one aim to assess the effect of ITM on depression in HF-patients and received alerts were treated by the telemedical center where physicians ensured temporary medical support while the main responsibility for patient's therapy was taken by their primary care physicians. Thus, our study assesses the effect of using ITM as follow-up modality on survival and hospitalization in HF-patients, and received alerts were treated by a dedicated cardiology staff to provide an immediate specialized intervention.

The available conflicting data on telemonitoring benefits in HF-patients may be partially related to the lack of a clear definition of alert management algorithm in some previously published studies. This study outcome highlights that the targets from applying HF-patients telemonitoring have ensued from providing an adequate reactive specialized response at the appropriate time. In other words, what could be the benefits from monitoring patient without anticipating an appropriate immediate intervention when requested. That's why these study results are in line with TIM-HF2 trial where the cardiologist was also responsible for treating alerts (3). Finally, providing a close monitoring associated with early specialized intervention seems the key element to figure out the benefits of telemonitoring in HF-patients.

To conclude, patient's adherence, fast reaction and appropriate specialized intervention are the pivotal parameters to enhance telemonitoring efficiency and improve the following-up process of HF-patients. The transient increase in diuretic dose at early stage of the congestive syndrome makes possible to avoid acute decompensation. This study result represents an important progress in the out-of-hospital care of HF-patients, thereby ITM may deserve better than the actual Class IIb recommendation proposed by the European experts (14).

### Limitations

The retrospective study design pooling two different cohorts enrolled over different time span may predispose to selection bias. Data were derived from two cohorts conducted over different periods of time which leads to potentially important confounder, the pharmacological treatment. As predicted, using ARNIs, ACEi, ARB and ivabradine were more commonly observed in the ITM-group. Nevertheless, adjusted multivariable and sub-group analyses have demonstrated the positive effects of ITM on unplanned hospitalization and all-cause mortality rate independently from the medical therapy (16, 17). Also, we mention the absence of a control group in the most recent cohort. The absence of significant association on cardiovascular mortality is most probably related to the low prevalence of the observed cardiovascular events which limits the power of the study to detect a statistical difference. However, a trend for a significant negative association seems to be likely. Lastly, the duration of follow-up in ITM-group was shorter than that in STM-group and SC-group, respectively (this difference is explained by the percentage of patients included in ETAPES program in 2020 and still under Telemonitoring at the date of analysis). We also mention a slight decrease in the received number of alerts during Spring 2020 in association with COVID-19 pandemic in France and the ensuing lock-down.

## Conclusion

To conclude, simple daily remote monitoring of weight and symptoms associated with specialized healthcare givers support for an early cardiologist intervention in HF-patients may reduce the rate of unplanned hospitalization and all-causes of mortality. Expanding ITM implementation in a large HF-population may help to decrease the huge burden of this disease on economic and public health systems.

## Data Availability Statement

The raw data supporting the conclusions of this article will be made available by the authors, without undue reservation.

## Ethics Statement

Ethical review and approval was not required for the study on human participants in accordance with the local legislation and institutional requirements. The patients/participants provided their written informed consent to participate in this study.

## Author Contributions

MG, RI, AM, and JR contributed to conception and design of the study. MG and RI organized the database. AM and VN performed the statistical analysis. AM wrote the first draft of the manuscript. PF, MR, RI, and JR wrote sections of the manuscript. All authors contributed to manuscript revision, read, and approved the submitted version.

## Conflict of Interest

MG has received research grants and speaker honorarium from Chronic Care Connect/Air Liquide healthcare. LL is an employee of Air Liquide Santé International and shareholder in Air Liquide. MR is an employee of CDM e Health a subsidiary of Air Liquide. The remaining authors declare that the research was conducted in the absence of any commercial or financial relationships that could be construed as a potential conflict of interest.

## Publisher's Note

All claims expressed in this article are solely those of the authors and do not necessarily represent those of their affiliated organizations, or those of the publisher, the editors and the reviewers. Any product that may be evaluated in this article, or claim that may be made by its manufacturer, is not guaranteed or endorsed by the publisher.
