# Supplementary material for: Benefits of Interventional Telemonitoring on Survival and Unplanned Hospitalization in Patients With Chronic Heart Failure
Source: Front Cardiovasc Med. 2022 Jul 14;9:943778. doi: 10.3389/fcvm.2022.943778 (PMC9332912; doi:10.3389/fcvm.2022.943778)
Supplement: Supplementary file 1 [file Table_1.DOCX]

**Supplementary material**

**Supplementary**

**Table 1**: Adjusted multivariate analysis investigating the association between interventional telemonitoring (ITM) without ARNIs intake and unplanned hospitalization compared to other study groups.

|  | OR_a_ | 95%CI | p-value |
| --- | --- | --- | --- |
| Age | 0.991 | [0.970-1.012] | 0.391 |
| Hypertension | 1.155 | [0.692-1.927] | 0.581 |
| Diabetes mellitus | 1.283 | [0.738-2.229] | 0.377 |
| Ischemic-HF | 1.143 | [0.692-1.889] | 0.600 |
| LVEF | 0.989 | [0.967-1.012] | 0.338 |
| Ivabradine use | 0.458 | [0.174-1.208] | 0.115 |
| (ACEi, ARB) | 0.539 | [0.293-0.992] | 0.047 |
| Ambulatory process | 0.971 | [0.361-2.615] | 0.954 |
| Groups  -STM-group  -ARNIs-ITM-group (n=77)  -NoARNIs-ITM-group (n=143) | 0.861  0.293  0.321 | [0.472-1.573]  [0.150-0.575]  [0.144-0.719] | 0.627  <0.001  0.006 |

**STM-group=standard telemonitoring-group. ITM-group=interventional telemonitoring group. BMI= body mass index. HF=heart failure. LVEF=left ventricular ejection fraction. ACEi= Angiotensin converting enzyme inhibitor. ARB: Angiotensinogen 2 receptor antagonist. ARNIs: Angiotensin receptor-neprilysin inhibitors.*

**Supplementary Table 2**. Adjusted multivariate analysis investigating the association between interventional telemonitoring (ITM) without ARNIs intake and all-cause mortality compared to other study groups.

|  | OR_a_ | 95%CI | p-value |
| --- | --- | --- | --- |
| Age | 1.034 | [1.002-1.067] | 0.038 |
| Hypertension | 0.549 | [0.270-1.117] | 0.098 |
| Diabetes mellitus | 1.294 | [0.614-2.730] | 0.498 |
| Ischemic-HF | 0.894 | [0.453-1.764] | 0.747 |
| LVEF | 0.957 | [0.926-0.988] | 0.007 |
| Ivabradine use | 0.441 | [0.094-2.076] | 0.300 |
| (ACEi, ARB) | 0.456 | [0.213-0.978] | 0.044 |
| Ambulatory process | 0.964 | [0.247-3.764] | 0.958 |
| Groups  -STM-group  -ARNIs-ITM-group (n=77)  -NoARNIs-ITM-group (n=143) | 1.251  0.264  0.237 | [0.585-2.675]  [0.097-0.718]  [0.062-0.898] | 0.564  0.009  0.034 |

**STM-group=standard telemonitoring-group. ITM-group=interventional telemonitoring group. BMI= body mass index. HF=heart failure. LVEF=left ventricular ejection fraction. ACEi= Angiotensin converting enzyme inhibitor. ARB: Angiotensinogen 2 receptor antagonist. ARNIs: Angiotensin receptor-neprilysin inhibitors.*
